# Yoga-Nidra as a mental health booster: A narrative review

**DOI:** 10.1016/j.jaim.2023.100842

**Published:** 2023-12-05

**Authors:** Khushboo Nayak, Kedarmal Verma

**Affiliations:** a National Institute of Ayurveda Jaipur, India; b School of Humanities and Social Sciences, Cognitive Experimental Laboratory, Indian Institute of Technology Indore, India

**Keywords:** Yogic sleep, Yoga-nidra, Intervention, Therapy

## Abstract

Mental health disorders are treated with all the available advanced health techniques. The pioneers of Indian philosophy, sages, saints, and yogis, through their experiences and rational explanations, expressed the importance of yoga, and their treatment effects. Yoga-Nidra (YN), one of a particular forms of yoga, is described as a simple and precise way of dealing with mental disorders. The use of YN as an intervention has been reported to reduce anxiety, anger, depression, post-traumatic stress disorder (PTSD), and other different kinds of psycho-physiological abnormalities. In addition to the role of Yoga-Nidra as an intervention tool, it also brings relaxation to the mind and brain, mental catharsis, a positive attitude, self-improvement, and personality refinement. At the same time, YN contributes to boost concentration, memory, and other cognitive capacities, including attention, and thoughts.

Because of its important therapeutic contribution to psychological well-being and mental health, it is currently used as a therapy and medical intervention. Yoga-Nidra and other yogic practices will play an important role in treating mental, physical, and psychological problems and improving cognitive abilities, and will help to connect with oneself.

## 1 Introduction

**Fig. 1:**
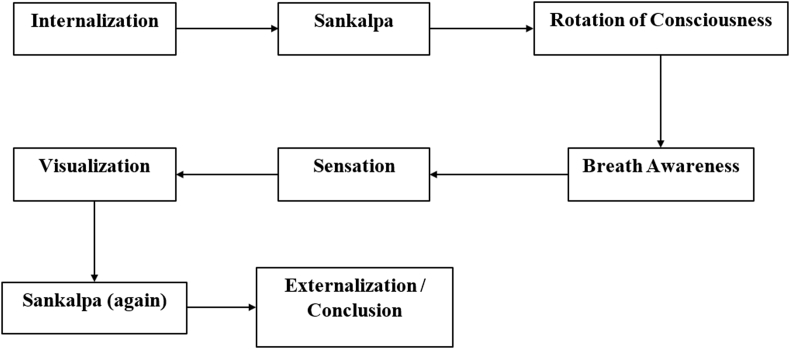
Different stages of yoga-nidra.

Throughout history, the sages have imparted knowledge to global society with the intention of promoting the well-being of humanity. The ancient traditions of India, such as the Vedas, Upanishads, and Bhagavad Gita, contain numerous scientifically validated practices aimed at promoting holistic human well-being. Atharvaveda explains the notion of the mind as the fundamental element of consciousness, serving as the internal source of knowledge, a channel for the acquisition of knowledge, the facilitator of hypnotic states, the guardian of will, the catalyst for emotional experiences, the impetus for motivation, and the locus of consciousness. In the same vein, the Upanishadas also offer comprehensive elucidation about the fundamental tenets of perception, cognition, awareness, and recollection. Furthermore, many states of consciousness are delineated, encompassing wakefulness, dreaming, deep sleep, and *samadhi* (see Fig. 1).

The necessity of exerting control over the mind arises from its fickle tendencies. Numerous methodologies, as advocated by the enlightened thinkers of ancient India, are cited for this purpose, with notable emphasis placed on the practice of yoga and pranayama. The pranayama and yoga practices encompass the regulation of bodily functions, management of sensory perceptions, and suppression of innate cravings, which serve as the root causes of both physical and mental maladies. By diligently practicing these yogic approaches, individuals might observe both qualitative and quantitative transformations in their psychological and physiological processes.

The concept of human mental health can be characterized as a condition of overall wellness, wherein an individual possesses the ability to effectively manage the various stressors encountered in their life and exhibit a high level of productivity in their occupational pursuits. The World Health Organization (WHO) delineated an all-encompassing health action plan spanning from 2013 to 2020, which encompasses four primary objectives. These objectives encompass proficient leadership and governance, mental health initiatives at the community level, promotion and prevention of mental health, utilization of information technology systems, and research pertaining to mental health.

The worldwide disease burden is believed to be influenced by neuropsychiatric disorders, which are responsible for approximately 14 % of its prevalence [1]. Numerous factors contribute to the challenges faced by individuals with mental disorders in accessing assistance and receiving appropriate treatment. Furthermore, the presence of various comorbidities poses challenges in accurately forecasting the prognosis of individuals, underscoring the significance of mental health in this context.

The purpose of this narrative review is to evaluate and consolidate therapeutic effects of Yoga Nidra (YN). The review explains Yoga Nidra effects on mental health, and and concludes with possibilities for future research. This review helps experts find new research areas that can improve mental health.

## 2 Search strategy

The present narrative overview employed a comprehensive literature search to identify relevant scholarly literature pertaining to the connection between yoga nidra and mental health. The search undertaken in this article encompassed the utilization of well-established databases like PubMed, Scopus, and Google Scholar. The aim was to ascertain scholarly articles that underwent peer review and were published in the last 25 years. The process of selecting search terms and keywords has been carried out with careful attention to include all aspects of the subject matter, including synonymous phrases, variants, and related concepts. The incorporation of Boolean operators, specifically AND enabled the establishment of logical relationships between search words. The approach utilized for conducting the search is meticulously documented and published in adherence to established protocols, assuring transparency, and facilitating the replication of the process.

The search terms employed in the PubMed search encompassed "yoga nidra" AND "mental health." The search yielded a total of 16 publications, out of which 11 were considered relevant to the specified keywords. A Scopus search using identical terms yielded a total of 13 articles, of which 11 articles were found to be directly relevant to the keywords employed. Of the 29 papers that were discovered, only 17 articles were deemed suitable for inclusion in the synthesis of this current narrative review article. This present review will exclusively consider journal articles written in the English language that directly focus on yoga nidra as an intervention. These selected articles were synthesized to generate the content for the review. The publications considered in this study encompass various types of Yogas that incorporate yoga-nidra while excluding those articles solely focused on yoga without the inclusion of yoga-nidra. This narrative review encompasses a comprehensive compilation of 35 studies. In addition to the total articles, 17 were included in the PubMed and Scopus databases for indexing purposes. The remaining 18 papers were sourced from grey research databases, specifically Google Scholar, as they were predominantly referenced in the primary publications. Table 1 presents a comprehensive compilation of the related information pertaining to the articles encompassed within this evaluation.

All study studies were thoroughly examined, regardless of their methodological variations, as this present narrative review aims to explore the significance of yoga-nidra in addressing mental health concerns. All the papers that were searched underwent a thorough evaluation process, which involved assessing their titles and abstracts to determine their suitability for inclusion in the final synthesis for the present study. The publications underwent a comprehensive evaluation, comparison, and synthesis to ascertain the significance of yoga-nidra meditation in enhancing mental well-being.

### 2.1 Search outcome

This narrative review encompassed a total of 35 studies, of which 17 were specifically examined to assess the impact of yoga nidra on mental health. Out of the total of 17 research analyzed, there were 5 reviews, 4 randomized controlled trials, 1 study utilizing mixed methods, 1 clinical trial, and 9 studies employing pre- and post-intervention assessments of yoga nidra practice. Among the 17 papers analyzed, a subset of 5 studies encompassed patients, with 3 of these studies specifically focusing on female participants exhibiting menstrual abnormalities, while the remaining 2 studies concentrated on persons diagnosed with post-traumatic stress disorder (PTSD). The remaining 12 trials encompassed a sample of persons who were in good health and ranged in age from 13 to 66 years. This study encompasses a comprehensive analysis of research conducted between 1998 and 2023, with a specific focus on articles pertaining to yoga nidra from 2008 to 2023. The research conducted assessed many psychological variables, including stress, anxiety, sadness, well-being, sleeplessness, somatoform symptoms, and other factors that are linked to aberrant somatic disorders. All these measurable variables have the capacity to serve as direct or indirect indicators of human mental health.

## 3 Yoga-Nidra and its Importance

Yoga-Nidra, often referred to as yogic sleep, conscious sleep, restless sleep, dynamic sleep, psychic sleep, or profound relaxation, is a remarkable mental condition that exists between wakefulness and sleep [2]. People who advocate for it claim that it is suitable for everyone, from people who have never tried yoga before to seasoned yogis who have been doing it for years. Yoga nidra practitioners assert that the state of inner quiet and stillness that is created by the practice is both a helpful tool for the management of stress and a method for obtaining a better sensitivity to one's resolves, including the goal of achieving self-transformation, the goal of enhancing creativity, or the goal of improving one's learning ability [2]. Swami Satyananda Saraswati and his guru Sivananda Saraswati are widely recognized as the pioneers of the current practice of YN, owing to their dedicated practice of this technique in Rishikesh [3]. The Mindful Yoga teacher Anne Cushman says that the body-sensing journey [that I teach in Mindful Yoga] is also a form of yoga-Nidra, which is used in the Buddhist-Vipassana technique [4].

Yoga Nidra helps individuals relax and meditate to maintain a high level of awareness. This practice promotes physical relaxation, mental clarity, and inner peace via body assessment, breath awareness, and visualizations. This groundbreaking technology fell into disuse over the course of time, but it is making a comeback now that it is being used by people all over the world. This practice is being used as a treatment for important mental illnesses such as stress, depression, post-traumatic stress disorder, and various other psychophysiological abnormalities [5,2], and on its own or in conjunction with other yogic techniques, has a major impact on the development of human cognitive abilities such as memory, concentration, and attention [6]. The utilization of the yoga-nidra technique has been found to be efficacious in managing stress-related ailments such as hypertension [7,8], mitigating the effects of stress, anxiety, fear, anger, and depression, sleep disorders [9,10,11]. The yoga-nidra technique also helps to achieve a state of equilibrium and overall welfare [12,13,14,15]. Many people think of YN as just a way to sleep which is a misconception, rather, YN is one such effective technique that not only provides mental relaxation but also physical relaxation. Yoga-Nidra also enhances our well-being and encourages our minds to concentrate. Researchers have agreed that yoga-nidra has a positive effect on mental health problems. Unlike ordinary sleep, YN entails a practice in which the practitioner achieves a state of slumber while maintaining full consciousness. During this practice, the body enters a state of sleep while the mind stays alert and receptive to the guidance provided by the instructor. Yogic sleep is a unique state of human consciousness that has the potential to induce deep relaxation across all dimensions, including the physical, mental, and emotional aspects [2]. This condition is sometimes described as the "hypnotic state," characterized by a transitional phase between sleep and awake [16,17].

## 4 Yoga Nidra and Mental Health Indices

Over the course of the past few years, it has been observed that the frequency of mental diseases among adults in a variety of regions across the globe has been progressively rising. A variety of psychological and physiological anomalies can each give rise to a particular type of mental disorder. The current treatments employed for addressing these conditions encompass both psychotherapy and pharmacological interventions [18]. There are multiple therapeutic techniques available for the management and treatment of mental health disorders. Nevertheless, patients frequently choose alternative approaches for a variety of reasons, such as experiencing adverse effects from medicine, insufficient response to conventional treatment, the financial burden of psychotherapies, or purely based on their individual preferences. A plethora of research papers have shown compelling data on the significant effectiveness of these tactics in the therapeutic intervention of mental disorders. Contemplative practices, also known as nonpharmacological techniques, are devoid of any adverse side effects, or if present, their magnitude is minimal. Furthermore, patients can engage in these practices independently following the provision of tailored recommendations. It is worth noting that contemplative practices have gained widespread international acceptance [19].

This review only focused on the meditative practice of YN and its impact on addressing mental health concerns. Yoga-Nidra is a form of guided meditation wherein a proficient practitioner facilitates individuals in attaining a meditative state through the utilization of images, music, and several other techniques. The efficacy of this remarkable meditation practice has been demonstrated in alleviating several psycho-physiological abnormalities, including stress, anxiety, rage, well-being, and post-traumatic stress disorder [20,12,21,22,23,24,25]. This therapy is currently being employed due to its potential to significantly impact heightened sensations of relaxation, tranquility, and overall mental and physical well-being in individuals.

The presence of mental loads within the academic environment has been noted. One of the studies examined the mental health status of faculty members and identified that academic stress can be alleviated with YN practice. Nevertheless, upon analyzing the demographic data, it was determined that there were no statistically significant disparities identified among the categories, apart from variations in the level of educational attainment. One study recruited individuals who were in good health, as well as individuals who were taking medication for diabetes, hypertension, and heart disease [26]. Similarly, a limited number of studies conducted on college students (14) and the general population (7) have indicated a significant reduction in stress and anxiety levels following the practice of YN. A separate investigation was conducted on adolescents, focusing on the period of transition between developmental phases, which has been associated with the potential emergence of mental health disorders. A study conducted by researchers discovered that the use of YN has been shown to have a positive impact on various aspects of adolescents' general well-being, as well as their cognitive and emotional states [24]. Mental health conditions are not just impacted by psychological variables; rather, biological elements can also play a significant role in their development and manifestation. Mensural irregularities have the potential to exert a detrimental impact on the mental well-being of women, either through direct or indirect means. Two distinct randomized controlled trials (RCTs) investigated the utilization of YN in conjunction with advanced medical interventions within the field of medical science. The findings of these trials indicate that this combined approach has been employed for the treatment of various psychosomatic disorders, including menstrual irregularities [27] and somatoform symptoms [28,29], specifically those arising from menstrual disorders in women. Previous studies have demonstrated that the implementation of YN has been effective in mitigating psychological distress, specifically depression and anxiety, which might arise due to irregularities in the menstrual cycle [[21], [28], [29], [30]]. Mental health concerns extend beyond the general population, as healthcare personnel also encounter a significant burden of mental health difficulties. Various adverse outcomes might be experienced by individuals, including physical discomfort, disruptions in sleep patterns, fatigue, absenteeism, and diminished levels of satisfaction [31]. Richard Miller created another intervention technique called Integrative Restoration (iRest) Yoga Nidra [13], like the practice of yoga nidra. One of the three intriguing research, as mentioned in the present review, examines the use of yoga nidra practice in healthcare workers to evaluate their levels of mindfulness, pain perception, and sleep patterns [32]. The results suggest that there is no statistically significant difference observed in the average ratings of pain, tiredness at baseline, and follow-up assessments. Nevertheless, there was a notable increase in the degree of mindfulness observed among healthcare practitioners after their involvement in an 8-week structured iRest Yoga Nidra intervention [23,25]. Stankovic (2011) conducted a study examining the effects of iRest on combative military survivors. The findings revealed a reduction in anger, anxiety, and emotional responsiveness, accompanied by an increase in feelings of calmness, tranquility, self-awareness, and self-confidence following the iRest intervention [23]. A study was conducted to examine the impact of iRest Yoga Nidra and acupuncture on psychological distress and depression among combat veterans [25].

Most investigations on yogic interventions have focused on evaluating enhancements in psycho-physiological behavior. But the specific neurological response to the yoga nidra intervention at the level of the brain remains unclear and has not been extensively investigated. A newly published study revealed notable alterations in brain activity after using yoga nidra. The investigators observed a decrease in the alpha and beta frequencies among the participants in the therapy group during their study. The discovery suggests that the inhibition of alpha waves offers a significant understanding of the cognitive mechanisms involved in the brain's preparation for integrating sensory information in a coordinated manner, hence promoting heightened attention [22]. A notable constraint of this study was the incorporation of additional yogic practices, namely Nadi-Shuddhi Pranayama and Nine-Centre Meditation, with YN. Consequently, the isolated impact of yoga nidra was not comprehensively elucidated.

In recent times, the global population has been confronted with the widespread COVID-19 epidemic, resulting in a multitude of mental health challenges including stress, melancholy, anxiety, and sleep disturbances. During the global crisis, a comprehensive lockdown and other limitations were implemented worldwide. Consequently, the management and alleviation of mental health disorders through pharmaceutical interventions have been shown to be challenging, necessitating the exploration and implementation of non-pharmaceutical approaches. Extensive research has been conducted to address these challenges through the utilization of yogic practice. However, it is worth noting that just a single study has examined the impact of yoga nidra and relaxation music in alleviating feelings of sadness, anxiety, depression, and insomnia among frontline health professionals [33]. The researchers discovered that YN demonstrates more efficacy compared to relaxation to music in decreasing various mental health-related variables.

Because YN is an excellent, easy, and cost-effective interventional approach that is also easily implementable, it may be used for the prevention of many psychosomatic diseases of the body. This is one of the reasons why Yoga Nidra is one of the most popular forms of alternative medicine today.

## 5 Procedure of Yoga-Nidra

The development of Yoga-Nidra is attributed to Swami Sri Satyananda Saraswati of the Yoga School of Munger in Bihar, who has provided a concise and accessible explanation of this practice. According to Swami Satyananda (1998), YN is a yogic technique that involves the internalization of one's awareness. During this practice, the individual steadily withdraws from external stimuli, ultimately reaching a condition when only the aural sensory channel is active [3]. Yoga-Nidra is a safe, straightforward, accessible, and cost-effective method for mitigating stress and addressing various psycho-physiological issues [7].

The practice of Yoga-Nidra encompasses eight stages, depicted in Fig. 1. These stages include the preparatory stage, Sankalpa, rotation of consciousness stage, breath awareness stage, sensation phase, visualization, Sankalpa (revisited), and the final externalization or conclusion phase. These stages guide the practitioner progressively towards the attainment of a remarkable state [8].

The practice of Yoga Nidra commences by engaging in preparatory measures, which involve assuming a comfortable supine posture and establishing a constructive intention. Shifting focus towards bodily awareness, deliberate attention is methodically focused on individual body parts with the aim of alleviating tension and fostering a state of relaxation. The practice of breath awareness enables individuals to study their innate breathing patterns, resulting in a state of mental tranquility. The act of establishing a *Sankalpa*, which refers to a constructive resolution, serves to initiate a process of profound change inside the subconscious mind. The utilization of guided visualization enhances the engagement of the world of imagination and creativity. Sensory awareness entails the process of seeing emotions and sensations without any form of connection or judgment. Ultimately, the procedure culminates in a re-establishment of consciousness, delicately guiding individuals back to the current instant and their environment [17,2]. Yoga Nidra is a practice that offers significant benefits in terms of rejuvenation, stress reduction, and self-exploration. It serves as a powerful means to attain inner peace and facilitate personal growth.

## 6 Future direction

The limitations observed in the examined studies include a very small sample size, the absence of a suitable sampling methodology, and a lack of blinding in treatment grouping (Table 1). The experiments conducted are hindered in drawing definitive results due to the restricted number of individuals assigned to each group. All measurements collected for the purpose of assessing mental health were based on self-reports provided by the participants, with only one research including an evaluation of brain activity. The limitations of reviewed studies may serve as prospective avenues for future research, with the aim of promoting the significance and effectiveness of yoga nidra as a therapeutic practice for mental health recovery.

**Table 1:** Summary of available research literature.

Title of the Paper	Nature of Research Article	Authors	Year	Study Population	Study Design	Type of Intervention	Duration	Results
The Origin and Clinical Relevance of Yoga Nidra	Narrative Review	Pandi-Perumal et al.	2022	NA	NA	NA	NA	Summarize the basic steps of yoga-nidra and its physiological and psychological effects
The Impact of Yoga Nidra and Seated Meditation on the Mental Health of College Professors	Original Research Article	Ferreira-Vorkapic et al.	2018	60 healthy adults of age between 30 and 55 years	Pretest-posttest: anxiety, stress, and depression were evaluated	Yoga-Nidra and Seated Meditation	3 months; Sessions: 45–50 min	Yoga-Nidra and seated meditation both helps in reducing the anxiety and stress level in college professors
Effect of Yoga-Nidra on Adolescent's Well-being: A Mixed Method Study	Research Article; Short Communication	Vaishnav et al.	2018	36 Adolescents; aged 13–15 years	Mixed Method; Pre- and Post-intervention	Yoga-Nidra	1-month; Sessions: 30 min daily for 3 days a week	The practice of yoga nidra has been shown to be helpful in increasing several facets of adolescents' overall well-being.
Psycho-Biological Changes with Add on Yoga Nidra in Patients with Menstrual Disorders: a Randomized Clinical Trial	Original Research Article	Rani et al.	2016	100 Women suffering from menstrual irregularities; aged between 18 and 45 years	randomized controlled trial	Yoga-Nidra	Sessions of 30–35 min per day, five days a week, led by a certified Yoga Therapist who specializes in Yoga Nidra for the first 3 months, the next 3 months continue the same at their homes (total 6 months intervention)	The psychological discomfort that relates to menstruation abnormalities may be successfully resolved by the practice of Yoga Nidra, which can be an effective form of practice.
Effectiveness of Integrative Restoration (iRest) Yoga Nidra on Mindfulness, Sleep, and Pain in Health Care Workers	Original Research Article	Livingston, & Collette-Merrill	2018	22 healthy employee volunteers; ages 18 and 65 years	pre-/post intervention descriptive survey	iRest Yoga Nidra	eight 1-h weekly iRest Yoga Nidra sessions	The results of the investigation demonstrated that participants' levels of mindfulness significantly increased after participating in a directed iRest Yoga Nidra program for 8 weeks.
Six-month trial of Yoga Nidra in menstrual disorder patients: Effects on somatoform symptoms	Original Research	Rani et al.	2011	A total of 150 female subjects were included suffering from menstrual irregularities; aged between 18 and 45 years	randomized controlled trial; pre-/post intervention	Yoga Nidra	(Yoga classes) consisted of a 35-min/day, five days in week intervention with Yoga Nidra	The results indicate that somatoform symptoms in patients with menstrual disorders can be decreased by learning and applying a program based on Yogic intervention (Yoga Nidra).
Yoga Nidra as a complementary treatment of anxiety and depressive symptoms in patients with menstrual disorder	Original Research	Rani et al.	2012	A total of 150 female subjects were included suffering from menstrual irregularities: aged 18–45 years	randomized controlled trial; pre-/post intervention	Yoga Nidra	Initially, all the patients with menstrual irregularities were recruited irrespective of the severity of anxiety or depressive symptoms. Following randomization, a psychological assessment of all patients was done to assess the severity of anxiety and depressive symptoms by applying the following tools (HAM-A and HAM-D). All the patients with clinically significant or non-significant anxiety or depressive symptoms were re-evaluated after six months	Patients who were presented with mild to severe anxiety and depression symptoms had a substantial improvement after participating in the 'Yoga Nidra intervention. Patients who were previously diagnosed as having significant anxiety and depression symptoms have not shown any signs of improvement.
Detecting Stress Level of Students Using Brain Waves Reducing It Using Yoga Therapy	Original Research	Rathi et al.	2022	40 students who are physically fit are selected and grouped into 20 Treatment groups and 20 control group	pre-/post intervention	Yoga Nidra, Nadi-Shuddhi Pranayama	about the period of 3–6 months	When students of the Treatment Group Practice Yoga Nidra and Nadi-Shuddhi Pranayama get together, there is a significant improvement in the level at which they can learn and recall the material. The academic marks of the group that was given treatment have seen a considerable improvement.
Efficacy of Yoga Nidra on Depression, Anxiety, and Insomnia in Frontline COVID-19 Healthcare Workers: A Pilot Randomized Controlled Trial	Original Research	Gunjiganvi et al.	2023	A total of 79 healthcare workers were randomly divided into two groups: (1) Relaxation-to-Music (n = 40) and (2) Yoga Nidra (n = 39).	open-label randomized trial	Music, and Yoga Nidra	both interventions were delivered through a YouTube platform and were to be done daily for 30 min during the healthcare workers' 2-week duty periods	For frontline COVID-19 medical professionals, practicing yoga nidra while at work was shown to be more useful than relaxing to music in terms of lowering symptoms of sadness, anxiety, and insomnia while they were on the job.
Mitigation of stress through yoga nidra (meditation) intervention	Original Research	Dwivedi	2021	200 respondents. The two groups were divided into the study: control and experimental groups	pre-/post intervention	Yoga Nidra	within an interval of one month	After the Yoga Nidra treatment was carried out, it was found that the participants experienced less stress than before the intervention. According to the findings, Yoga Nidra is an excellent method for reducing the negative effects of stress.
Exploring the uses of yoga nidra: An integrative review	integrative review	Musto, & Hazard	2023	NA	NA	NA	NA	Yoga Nidra was found to be effective in most of these studies.
Transforming trauma: a qualitative feasibility study of integrative restoration (iRest) yoga Nidra on combat-related post-traumatic stress disorder	Original Research	Stankovic	2011	16 male combat veterans (15 Vietnam War and 1 Iraq War) of mixed ethnicity, aged 41–66 years, suffering from posttraumatic stress disorder (PTSD).	Clinical trial	iRest Yoga Nidra	eight-week study examined the feasibility of offering weekly classes in Integrative Restoration (iRest)	Despite difficulties with mental focus and intrusive recollections, individuals who participated in the study and completed it reported improvements in their levels of relaxation, serenity, self-awareness, and self-efficacy, as well as a reduction in anger, anxiety, and emotional reactivity.
Psychological effects of yoga nidra in women with menstrual disorders: A systematic review of randomized controlled trials	systematic review	Kim, Sang-Dol	2017	NA	NA	NA	NA	There is evidence from two RCTs that yoga nidra may have favorable effects in terms of reducing psychological problems in women with menstrual disorders.
The Effect of Mindfulness and Acupuncture on Psychological Health in Veterans: an Exploratory Study	Original Research	Wheeler et al.	2018	328 Veterans enrolled in treatment at the IHW Program during the study period, 226 consented to participate in the research	pre-/post intervention	iRest Yoga Nidra with Acupuncture	iRest sessions were offered four times a week (of which one session each week was for women only); acupuncture sessions were offered three times a week.	The combined treatment was equally beneficial to independent of factors such as age, gender, or race. findings lend preliminary support for the extension of complementary and integrative health offerings including iRest and acupuncture to more Veterans Administration hospitals across the country to improve military mental health.
Yoga-nidra and hypnosis	Narrative Review	Hoye, & Reddy	2016	NA	NA	NA	NA	provide an overview of yoga-nidra, both its origins and current form of practice
Yoga Nidra as a Stress Management Intervention Strategy	Review	Dwivedi, & Singh	2016	NA	NA	NA	NA	The practice of Yoga Nidra is a potent method that may bring about full relaxation on all levels—physical, mental, and emotional.
A study on the impact on stress and anxiety through Yoga nidra	Original Research	Kamakhya Kumar	2008	80 students; 20–30 years of age	pre-/post intervention	Yoga Nidra	Practice time was 30-min with a duration of 6 months	Yoga nidra positively decreases the stress level of the male and female subjects

Most studies investigating the effects of YN interventions have been focused on individuals who are in good health. The implementation of yoga nidra intervention in individuals experiencing clinical distress has the potential to facilitate the utilization of therapy modalities that are accessible, affordable, and efficacious. Conducting comprehensive and extensive research on this efficacious intervention is of paramount importance. This necessitates incorporating many time points (e.g., 4, 8, or 12 weeks), a bigger sample size, and the inclusion of control groups, treatment groups, and clinical groups. The process of upgrading and confirming the results is crucial to ensure their accuracy and reliability. The following two major future implications could be best to understand the mechanism of yoga nidra meditation.

### 6.1 Population considerations

In the field of Yoga Nidra research, a fundamental strategy for enhancing the dependability and practicality of findings involves increasing the size of the sample. To improve the precision of Yoga Nidra studies, it is imperative that forthcoming research places a high priority on incorporating a diverse variety of demographic characteristics among participants. This encompasses a variety of criteria, such as age, gender, cultural heritage, and health-related concerns. To enhance the broader applicability of the study's results to a more heterogeneous population, it would be beneficial to broaden the inclusion criteria for participant selection. The utilization of longitudinal research, coupled with a larger sample size, has the potential to yield significant insights into the long-term effects of consistent Yoga Nidra practice. The acquisition of data from research endeavors with larger sample sizes will serve as the fundamental basis for conducting comprehensive meta-analyses and systematic reviews. These endeavors possess the ability to amalgamate data from multiple research studies, discern trends, and yield more precise estimations of effects. Consequently, our findings may provide a stronger foundation for formulating policy recommendations and guiding therapeutic approaches. Partnerships across research organizations can facilitate the conduct of multicenter experiments with large sample sizes. These studies possess the potential to investigate the replicability of findings across many contexts and individuals, thereby bolstering the reliability of outcomes and contributing to the validation of the efficacy of Yoga Nidra.

### 6.2 Electrophysiological perspectives

In the dynamic field of Yoga Nidra research, there exists a promising avenue for further exploration that entails doing a thorough examination of the electrophysiological aspects. The investigation of the neurological and physiological mechanisms behind Yoga Nidra holds promise for advancing our understanding of its effects on the mind-body connection.

The opportunity to uncover the complex cerebral mechanisms underlying the practice of Yoga Nidra exists with the application of advanced neuroimaging techniques including functional magnetic resonance imaging (fMRI), electroencephalography (EEG), and magnetoencephalography (MEG). These techniques will facilitate the monitoring and analysis of dynamic changes in neural activity, enabling researchers to identify with precision the specific brain areas and circuits that are impacted by the practice of Yoga Nidra.

An additional intriguing viewpoint pertains to the significance of brain connectivity among distinct cerebral regions, a phenomenon that has the potential for enhanced comprehension through the practice of Yoga Nidra. The investigation of neural network coherence and synchronization enables researchers to get insights into the collective functioning of brain regions during the process of skill acquisition. Gaining insight into these patterns of connectivity will serve to elucidate the coherence of psychological, cognitive, and autonomic processes.

A thorough electroencephalogram (EEG) investigation has the potential to unveil the mechanisms underlying the control of brain wave activity during the practice of Yoga Nidra. The impacts on awareness can potentially be discerned by the examination of different brain wave patterns, such as alpha, beta, theta, and delta waves. This information will elucidate the concepts of relaxation, meditation, and introspection in the context of Yoga Nidra. It is imperative to comprehend the electrophysiological markers associated with states of tranquility and alleviation of tension. Researchers have the potential to establish measurable parameters for assessing the impact of Yoga Nidra on the body's stress response mechanisms through the examination of alterations in brain oscillations, hormone levels, and autonomic responses.

The potential for future research inquiries on Yoga Nidra appears highly promising in shedding light on the electrophysiological mechanisms underlying its transformative advantages. Researchers can enhance their understanding of the intricate mind-body connections facilitated by Yoga Nidra through the utilization of advanced neuroimaging techniques. By investigating the complexities of neural connectivity within the brain, analyzing brain wave patterns, and identifying biomarkers associated with relaxation, researchers can make significant progress in comprehending this phenomenon.

## 7 Conclusion

In most of the research, the Yoga Nidra practice was discovered to be beneficial. However, there is also clinical heterogeneity in the sample groups, treatment session lengths, times, and durations in most of the research. Because of this, it can be tough to generalize on YN intervention based simply on the data obtained in the reviewed studies. The current paper was analyzed to have a better understanding of the effects that yoga nidra has on a variety of mental health disorders. In general, there is a need to conduct a greater quantity of research, particularly studies that have larger sample sizes and more rigorous experimental designs. Specifically, this opportunity exists for studies like these. The practice of YN shows promise as an effective, non-invasive, and pharmacologically-free therapy or adjuvant for a wide range of health concerns, especially those related to the mind. Yoga Nidra also has the added benefit of being able to alleviate stress.
